# Neurotrophic factors combined with stem cells in the treatment of sciatic nerve injury in rats: a meta-analysis

**DOI:** 10.1042/BSR20211399

**Published:** 2022-01-14

**Authors:** Kuang-Pin Liu, Wei Ma, Chun-Yan Li, Li-Yan Li

**Affiliations:** Institute of Neuroscience, Kunming Medical University, Kunming, Yunnan, China

**Keywords:** meta-analysis, neurotrophic factor, RAT, sciatic nerve injury, stem cells

## Abstract

Treatment of peripheral nerve regeneration with stem cells (SCs) alone has some limitations. For this reason, we evaluate the efficacy of neurotrophic factors combined with stem cell transplantation in the treatment of sciatic nerve injury (SNI) in rats. PubMed, Cochrane Library, Embase, WanFang, VIP and China National Knowledge Infrastructure databases were retrieved from inception to October 2021, and control experiments on neurotrophic factors combined with stem cells in the treatment of SNI in rats were searched. Nine articles and 551 rats were included in the meta-analysis. The results of meta-analysis confirmed that neurotrophic factor combined with stem cells for the treatment of SNI yielded more effective repair than normal rats with regard to sciatic nerve index, electrophysiological detection index, electron microscope observation index, and recovery rate of muscle wet weight. The conclusion is that neurotrophic factor combined with stem cells is more conducive to peripheral nerve regeneration and functional recovery than stem cells alone. However, due to the limitation of the quality of the included literature, the above conclusions need to be verified by randomized controlled experiments with higher quality and larger samples.

## Introduction

Development and maintenance of the nervous system depends on proteins called neurotrophic factors [1]. In mammals, the neurotrophic factor family originally included nerve growth factor (NGF), brain-derived neurotrophic factor (BDNF), neurotrophic factor (NT)-3 (NT-3), and NT-4/5, as these proteins were identified as factors related to neuron survival [2]. Subsequently, cholinergic neurotrophic factor (CNTF) and glial-derived neurotrophic factor (GDNF) were added to this family, as they were also shown to promote survival and differentiation of multiple target neurons in central and peripheral regions. Members of the neurotrophic factor family are widely expressed in the developing and mature central nervous system (CNS), especially during synaptogenesis, which involves the formation of synaptic structures, as well as signal transmission between presynaptic and postsynaptic neurons [3]. In addition, in the context of spinal cord injury, neurotrophic proteins can be delivered to the injured spinal cord to support the growth of many discrete neuron populations. For example, NGF promotes cholinergic release to local motor axons and mediates the regeneration of primary sensory axons after injury; BDNF secretion promotes bone marrow stromal cell transplantation to affect neurons and sensory axon regeneration and NT-3 (expressed in a similar pattern to BDNF) promotes regeneration of dorsal root neurons and ascending sensory neurons in the spinal cord [4].

Compared with the CNS, the peripheral nervous system (PNS) has a strong regenerative capacity; although, this regeneration is far from complete. In other words, functional recovery after injury rarely recovers to the pre-injury level. During development, the PNS is strongly dependent on nutritional stimuli, which affect the differentiation, growth, and maturation of neurons. This important nutritional stimulus is the neurotrophic factor family (NGF, BDNF, NT-3, and NT-4/5). Peripheral nerve injury disrupts normal function of sensory and motor neurons by destroying the integrity of axons and Schwann cells [5]. Sciatic nerve injury (SNI), one type of peripheral nerve injury, can lead to increased secretion of NGF, BDNF, CNTF, GDNF, and insulin-like growth factors (IGFs). Hollis et al. found that the application of BDNF, NT-3, or IGF-1 could improve the loss of motor neurons in neonatal mice with SNI [4]. Chen et al. showed synergistic effects of NGF, CNTF, and GDNF on the survival and growth of sensory and motor neurons, as well as functional recovery after SNI in rats [6]. However, researchers are still exploring treatments for nervous system injury. BDNF, NT-3, and NT-4 can be used as survival factors for human embryonic stem cells (hESCs). Indeed, by adding neurotrophic factors to hESC cultures, the clonal survival rate is increased to 36-times baseline values. This confirms that cooperation of hESCs with neurotrophic proteins plays a role in nerve regeneration [7]. Dong et al. used NT-3 gene transfection *in vitro* to increase the number of bone marrow-derived mesenchymal stem cells (BMSCs) in the spinal cord injury area. Moreover, they confirmed that NT-3 can promote the survival of BMSCs transplanted into the spinal cord injury area, and may enhance the therapeutic effect of spinal cord injury repair [8].

At present, most studies focus on neurotrophic factor-binding stem cells (SCs) in spinal cord injury, which provides clues for regeneration of the CNS. However, the regenerative ability of the PNS does not seem to match the level of target organ innervation, leading to slower functional recovery [9]. In recent years, scholars at home and abroad have attempted to combine neurotrophic factors with stem cells to treat SNI in rats as a less complex treatment method to facilitate recovery of peripheral nerve injury. Herein, we present a meta-analysis of related research to provide a reference for future studies.

## Methods

### Search strategy

Randomized controlled studies of neurotrophic factors combined with stem cells in the treatment of SNI in rats were searched in PubMed, Cochrane Library, Embase, WanFang, VIP, and China National Knowledge Infrastructure databases from inception to October 2021. Chinese and English search terms included peripheral nerve injury, SNI, stem cells, neurotrophic factors, NT-3/4/5, BDNF, GDNF, NGF, rats, randomized controlled trial (RCT), and meta-analysis.

### Inclusion and exclusion criteria

The inclusion criteria were defined as: (1.) Controlled experiment; (2.) A rat model with SNI, either left or right; (3.) Intervention measures are the use of neurotrophic factors combined with stem cells; (4.) Postoperative observation for no less than 2 weeks; (5.) Language limited to Chinese and English. Studies were excluded if one of the following existed: (1.) Other animal experiments and non-animal experiments in non-rats; (2.) Lack of data to extract outcome indicators.

### Data extraction and quality assessment

According to inclusion and exclusion criteria, two researchers screened the literature and extracted the data. If there were differences, the third researcher would help resolve the problem. The main contents of extracted data included the title of the article, first author, date of publication, and journal; basic information about the rats including numbers, genetic strains, modeling methods; and outcome indicators of the study: (1.) Sciatic nerve function index (SNFI), (2.) Muscle wet weight recovery rate, (3.) Electron microscopy observation, (4.) Neuroelectrophysiological detection. The Cochrane Manual was used to evaluate literature quality, including proper use and implementation of randomization, distribution concealment, and blind methods; data completeness, selective reporting of results. Evaluation results were recorded as ‘yes’, ‘no’, or ‘unclear’.

### Statistical analysis

Meta-analysis was performed using Review Manager (RevMan) version 5.3 statistical software provided by Cochrane Collaboration (2014). Mean difference (*MD*), standardized mean difference (*SMD*), and 95% confidence interval (*CI*) were used as effect analysis statistics. When the measurement methods or units of the same intervention effect were identical, *MD* was chosen; whereas, *SMD* was chosen for combined statistics when different measurement methods or units were used for the same intervention effect. A chi-square test was used to estimate the level of heterogeneity among studies, and heterogeneity among the results was assessed according to the size of *I^2^*; if the heterogeneity was considered to be large, such as *I^2^ >* 50%, a random-effects model was used for meta-analysis. However, if *I^2^* < 50% and the heterogeneity is small, meta-analysis was carried out using a fixed-effects model. Meta-analysis was repeated by eliminating low-quality studies or adopting different evaluation criteria and statistical methods. *P<*0.05 was considered to have statistical significance.

## Results

### Study selection and characteristics

**Figure 1:**
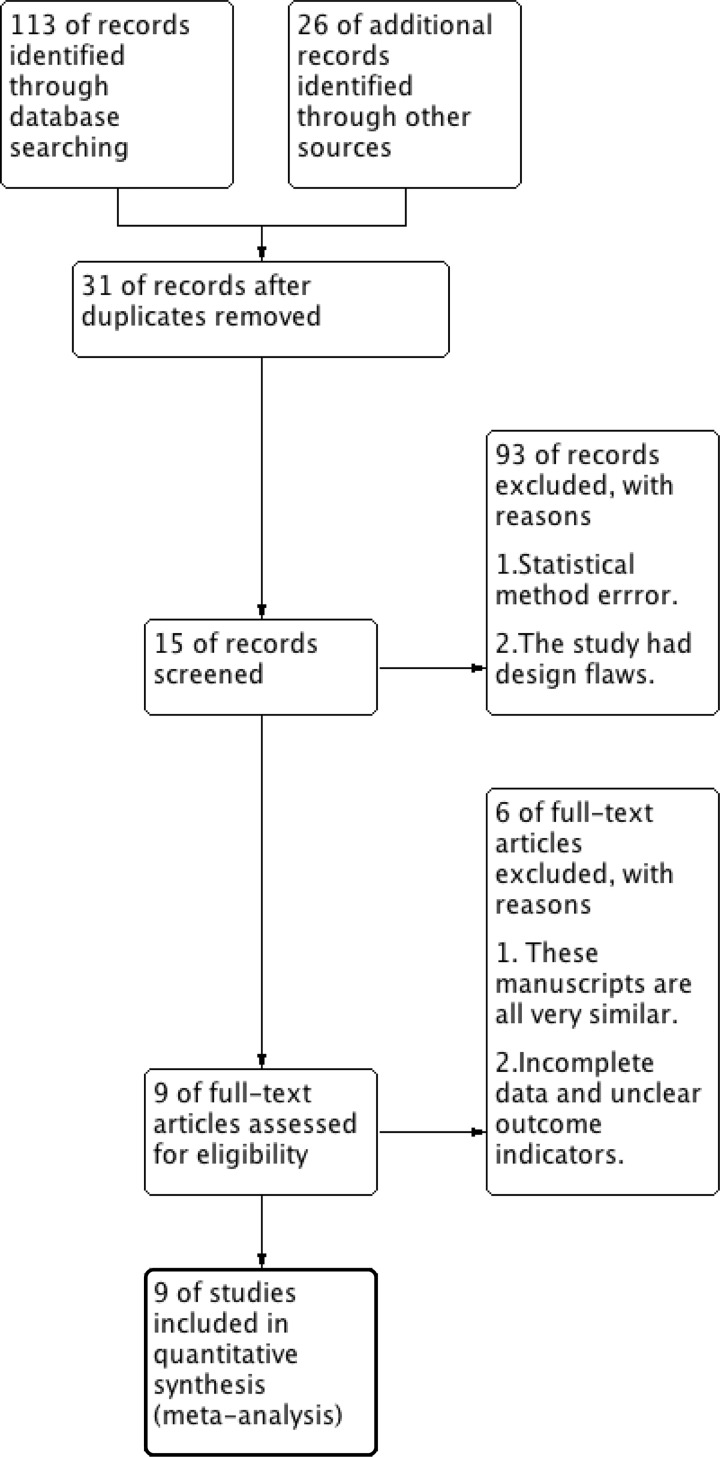
Flow diagram of the study selection process

Of a total of 139 related articles were retrieved, 15 were screened out by browsing the topics and abstracts. After researching and reading the full text according to relevant topics, 9 articles were finally included according to inclusion and exclusion criteria, 164 rats were included (Figure 1). The basic characteristics of the included literature are shown in Table 1, and the quality evaluation results are shown in Table 2.

**Table 1:** Summary of characteristics of the included studies

Year	First author	Strain	Model	Stem cell	Stem cell source	Modification	Number of cases (experimental group/control group)	Outcome indicators
2019	Zheng [10]	F344 rats	Electrical damage	ADSCs	Autogenous	GDNF-transfected	6/6	SFI
2018	Moattari [11]	Wistar rats	SNI	MSCs	Allogeneic	Direct injection with NGF	6/6	Amplitude, Latency, Medullated nerve fiber count
2017	Hei [12]	SD rats	SNI	Human UCB-MSCs	Allogeneic	BDNF-transfected	20/20	SFI
2015	Zhang [13]	SD rats	SNI	BMSCs	Allogeneic	CNTF-transfected	10/10	Myelin sheath thickness, Recovery rate of wet weight, Amplitude, Latency
2015	Yang [14]	SD rats	Electrical damage	ADSCs	Autogenous	GDNF-transfected	6/6	Myelin sheath thickness, Medullated nerve fiber count
2014	Shang [15]	SD rats	CCI	BMSCs	Autogenous	GDNF-transfected	10/10	SFI, Myelin sheath thickness, Medullated nerve fiber count, Recovery rate of wet weight
2014	Zhang [16]	SD rats	SNI	BMSCs	Allogeneic	BDNF-transfected	10/10	SFI, Myelin sheath thickness, Recovery rate of wet weight
2009	Zheng [17]	F344 rats	SNI	BMSCs	Autogenous	BDNF-transfected	10/10	SFI
2005	Lei [18]	SD rats	SNI	NSCs	unclear	Direct injection with NGF	4/4	Recovery rate of wet weight

Abbreviations: ADSC, adipose-derived stem cell; CCI, chronic constriction injury; human UCB-MSC, human cord blood mononuclear cell; MSC, mesenchymal stem cell; NSC, neural stem cell; SD rat, Sprague–Dawley rat; SNFI, sciatic nerve function index.

**Table 2:** Quality assessment of studies included in meta-analysis

Year	First author	Randomization	Distribution concealment	Blind methods	Data completeness	Selective reporting
2019	Zheng [10]	Yes	Yes	Yes	Yes	Yes
2018	Moattari [11]	Yes	Yes	Yes	Yes	No
2017	Hei [12]	Yes	Yes	Yes	Yes	Yes
2015	Zhang [13]	Yes	Yes	Yes	Yes	No
2015	Yang [14]	Yes	Yes	Yes	Yes	No
2014	Shang [15]	Yes	Yes	Yes	Yes	Yes
2014	Zhang [16]	Yes	Yes	Yes	Yes	No
2009	Zheng [17]	Yes	Yes	Yes	No	Yes
2005	Lei [18]	Yes	Yes	Yes	Yes	No

### Results of individual studies

#### SNFI

**Figure 2:**
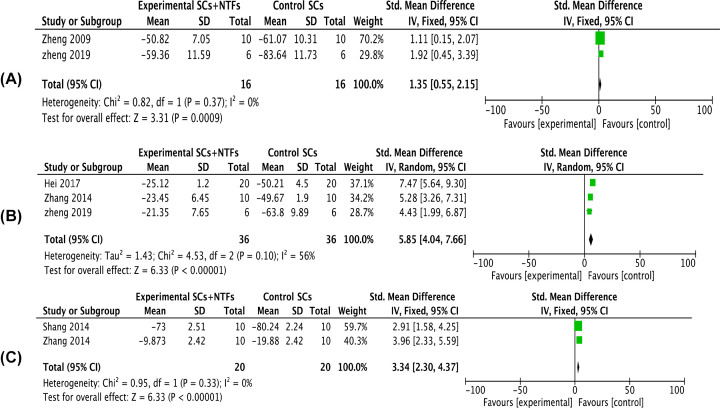
Forest plots of SNFI (**A–C**) Showed the meta-analysis of sciatic nerve index of the affected limb after stem cell treatment and stem cell combined neurotrophic factor treatment in rats with sciatic nerve injury at 2, 4, and 8 weeks, respectively. Experimental group: stem cells combined with neurotrophic factors treatment group; Control group: stem cells treatment group. Neurotrophic factor combined with stem cell in the treatment of sciatic nerve function index was significantly better than that in the stem cell treatment group, the difference was significant (*P*<0.05).

Two articles reported the SNFI of affected limbs 2 weeks after transplantation of neurotrophic factor combined with stem cells (*I^2^* = 0%). Meta-analysis using a fixed-effects model indicated that the SNFI of the combined treatment group was significantly better than that of the stem cell only group [*SMD* = 1.35, 95% *CI* (0.55, 2.15), *P*=0.0009] (Figure 2A). Three articles reporting the SNFI of affected limbs at 4 weeks after operation (*I^2^* = 56%) exhibited some heterogeneity. Meta-analysis using a random-effects model indicated that the SNFI of rats treated with neurotrophic factors combined with stem cells was significantly better than the stem cells only group [*SMD* = 5.85, 95% *CI* (4.04, 7.66), *P*<0.00001] (Figure 2B). Two articles reported the SNFI of affected limbs at 8 weeks after operation (*I^2^* = 0%). Meta-analysis using a fixed-effects model showed that the SNFI of the combination treatment group was significantly better than that of the stem cell therapy alone [*SMD* = 3.34, 95% *CI* (2.30, 4.37), *P*<0.00001] (Figure 2C).

#### Recovery rate of muscle wet weight

**Figure 3:**
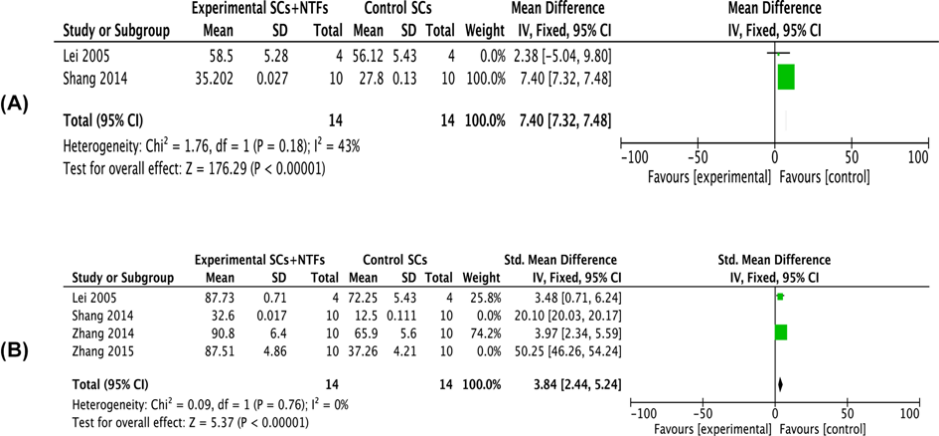
Forest plots of recovery rate of muscle wet weight (**A**,**B**) Showed the meta-analysis of recovery rate of muscle wet weight of the affected limb after stem cell treatment and stem cell-combined neurotrophic factor treatment in rats with sciatic nerve injury at 4 and 8 weeks, respectively. Experimental group: stem cells combined with neurotrophic factors treatment group; Control group: stem cells treatment group. Neurotrophic factor combined with stem cell in the treatment of recovery rate of muscle wet weight was significantly better than that in the stem cell treatment group, the difference was significant (*P*<0.05).

Two articles reported the recovery rate of muscle wet weight of affected limbs after 4 weeks of treatment with neurotrophic factor combined with stem cells (*I^2^* = 43%). Meta-analysis using a fixed-effects model indicated that the recovery rate of muscle wet weight in the combined treatment group was significantly increased compared with the stem cell treatment group [*MD* = 7.40, 95% *CI* (7.32, 7.48), *P*<0.00001] (Figure 3A). Four articles reported the recovery rate of muscle wet weight of affected limbs after 8 weeks of treatment with neurotrophic factor combined with stem cells (*I^2^* = 0%). Meta-analysis using a fixed-effects model showed that the recovery rate of muscle wet weight in the combined treatment group was significantly increased compared with the stem cell treatment group [*SMD* = 3.84, 95% *CI* (2.44, 5.24), *P*<0.00001] (Figure 3B).

#### Neuroelectrophysiological indicators

**Figure 4:**
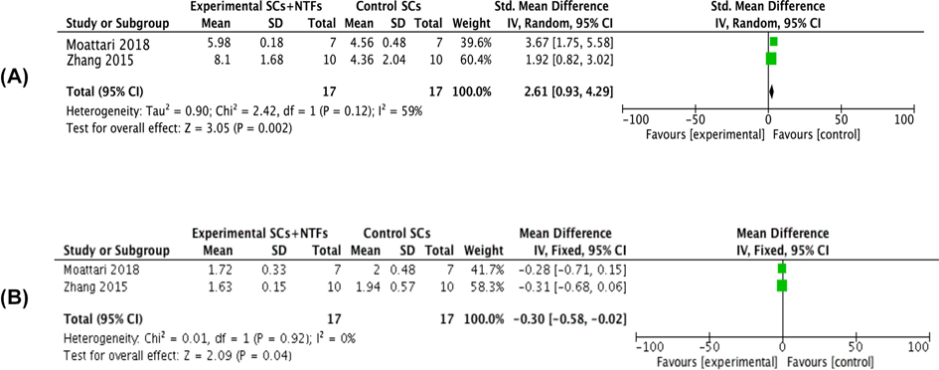
Forest plots of neuroelectrophysiological indicators (**A**) showed the meta-analysis of amplitude of nerve signal of the affected limb after stem cell treatment and stem cell combined neurotrophic factor treatment in rats with sciatic nerve injury at 8 weeks. (**B**) Showed the meta-analysis of latency period of nerve signal of the affected limb after stem cell treatment and stem cell combined neurotrophic factor treatment in rats with sciatic nerve injury at 8 weeks. Experimental group: stem cells combined with neurotrophic factors treatment group; Control group: stem cells treatment group. Neurotrophic factor combined with stem cell in the treatment of neuroelectrophysiological indicators were significantly better than that in the stem cell treatment group, the difference was significant (*P*<0.05).

Two articles reporting the amplitude of nerve signals in affected limbs at 8 weeks after treatment (*I^2^* = 59%) exhibited some heterogeneity. Meta-analysis using a random-effects model showed that the amplitude of nerve signals in the combined treatment group was significantly increased compared with the stem cell treatment group [*MD* = 2.61, 95% *CI* (0.93, 4.29), *P*=0.002] (Figure 4A). Two articles reported the latency of nerve signal in affected limbs at 8 weeks after treatment (*I^2^* = 0%). Meta-analysis using a fixed-effects model showed that there was no significant difference between the latency of nerve signals in combined treatment and stem cell treatment groups [*MD* = −0.30, 95% *CI* (−0.58, 0.02), *P*=0.04] (Figure 4B).

### Electron microscopy observations

**Figure 5:**
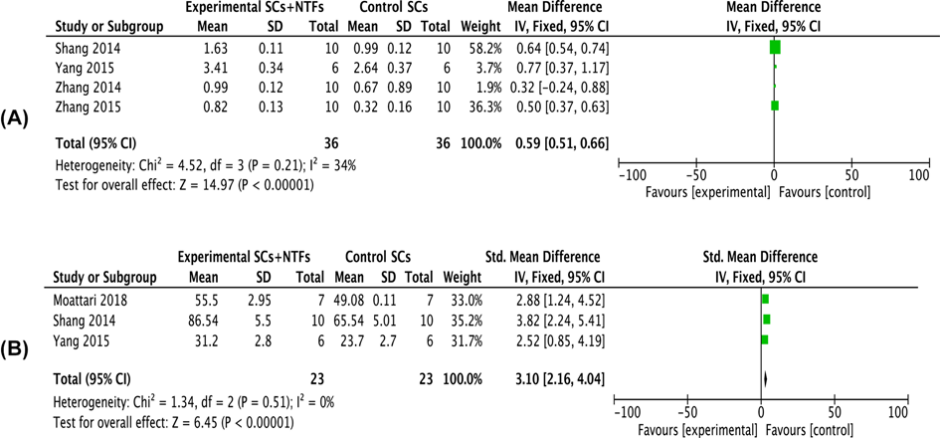
Forest plots of nerve transmission electron microscopy indicators (**A**) Showed the meta-analysis of thickness of myelin sheath of the affected limb after stem cell treatment and stem cell combined neurotrophic factor treatment in rats with sciatic nerve injury at 8 weeks. (**B**) Showed the meta-analysis of number of myelinated nerve fibers of the affected limb after stem cell treatment and stem cell combined neurotrophic factor treatment in rats with sciatic nerve injury at 8 weeks. Experimental group: stem cells combined with neurotrophic factors treatment group; Control group: stem cells treatment group. Neurotrophic factor combined with stem cell in the treatment of nerve transmission electron microscopy indicators were significantly better than that in the stem cell treatment group, the difference was significant (*P*<0.05).

Four articles reported myelin sheath thickness at 8 weeks after neurotrophic factor combined with stem cells treatment of SNI (*I^2^* = 34%). Meta-analysis using a fixed-effects model showed that myelin sheath thickness in the combined treatment group was significantly increased compared with the stem cell treatment group [*MD* = 0.59, 95% *CI* (0.51, 0.66), *P*<0.0001] (Figure 5A). Three articles reported the number of myelinated nerve fibers at 8 weeks after treatment (*I^2^* = 0%). Meta-analysis using a fixed-effects model showed that the number of myelinated nerve fibers in the combined treatment group was significantly increased compared with the stem cell treatment group [*SMD* = 3.10, 95% *CI* (2.16, 4.04), *P*<0.00001] (Figure 5B).

### Heterogeneity, sensitivity analysis

If *I^2^* < 50%, *P*<0.05, the heterogeneity is within a reasonable range. In the present study, there are two data *I^2^* > 50%, which are the SNIF of affected limbs at 4 weeks after treatment (*I^2^* = 56%) (Figure 2B) and the amplitude of nerve signals in affected limbs at 8 weeks after treatment (*I^2^* = 59%) (Figure 4A) demonstrated some heterogeneity; therefore, we performed sensitivity analysis, excluded each of the included studies one by one and re-analyzed the results similar to the original analysis (results not shown). In addition, considering that the heterogeneity is not very significant and there are few factors affecting these two indicators, it is not necessary to do subgroup analysis.

## Discussion

Axon regeneration and functional recovery after peripheral nerve injury remain clinical challenges. Currently, the use of cells, genes, and/or biological materials to treat peripheral nerve injury are promising approaches to repair [19]. However, there are still many problems for the application of biomaterials, such as immune rejection, uncertain degradation rates, toxicity of degradation products, limited sources of materials, and high costs [20]. In recent years, cell-based therapy for peripheral nerve injury has brought new breakthroughs in the field of regenerative medicine. In the PNS, stem cells can increase axon regeneration, myelin regeneration, and muscle preservation after SNI, indicating that stem cells play a beneficial role in nerve regeneration [21]. *In vivo* gene therapy studies have also shown satisfactory results [22]. Indeed, there is no doubt that the release of neurotrophic factors promotes axon regeneration and functional recovery after peripheral nerve injury [23]. Generally speaking, combining neurotrophic factors with stem cells as a somatic cell-based gene therapy has great potential for nerve repair and, thus, deserves further study.

Nine studies were included in this meta-analysis. The results showed that the SNFI at 2, 4, and 8 weeks after SNI was improved in rats treated with neurotrophic factor combined with stem cells compared with stem cells alone; moreover, limb function recovery of these rats was better. However, heterogeneity of SNFI at the fourth week may be related to the survival of transplanted cells in the nerve or the time required for peripheral nerve repair. After stem cell transplantation, individual differences lead to different adaptation mechanisms [24]. Notably, at 8 weeks, this heterogeneity disappeared, which may also be related to the different types of cells included in the study. As such, further studies are necessary to evaluate differences between different types of cells. The latest study by Zhang et al. [25] found that overexpression of BDNF and GDNF combined with BMSCs can promote peripheral nerve repair, and the SNFI value is better than that of BMSCs alone. However, the research data only disclosed the time point of 3 months after treatment. Different from other included literature, there is a lack of test results at 2, 4, 6, and 8 weeks after treatment, and the published data lacks standard deviation and mean value, it was not suitable for inclusion in our study.

Nerve electrophysiological detection can directly reflect signal transmission during nerve repair after peripheral nerve injury. Peak amplitude and incubation period of the neurotrophic factor-combined stem cell group were significantly increased compared with the stem cell treatment group, but there was some heterogeneity within the former group. Studies have shown that axonal injury and demyelination of different degrees can affect the electrophysiological detection indexes of the model [26], and the detection time points included in the study were all less than 12 weeks (the time point commonly used for electrophysiological detection after administration of clinical drugs to treat peripheral nerve injury). Electron microscopy showed that both myelin sheath thickness and number of myelinated nerve fibers were increased in the group treated with neurotrophic factor and stem cells compared with the group treated with stem cells alone after 8 weeks. Myelinated nerve fibers, which play an important role in signal transmission and protection of nerve cells, mainly exist in peripheral nerves. Effective myelin sheath regeneration is of great help to the recovery of peripheral nerve function. Notably, the recovery rate of muscle wet weight in the combined treatment group was also increased compared with the stem cell treatment group at 4 and 8 weeks after treatment. In the present study, meta-analysis indicated that the effect of neurotrophic factor combined with stem cells in the treatment of SNI was better than that of stem cells alone.

This meta-analysis has some shortcomings with regard to the whole analytical process, such as the small number of studies and small sample size of included experimental groups, and fact that some studies were not randomized, which may have a certain impact on the results. In addition, the half-life of neurotrophic factors, efficiency of viral vectors, and differences between stem cells should be included in future studies and analysis. Moreover, the modification of biomaterials in some articles can be a source of heterogeneity, which needs further analysis.

In conclusion, the present study is the first meta-analysis to evaluate the effect of stem cells combined with neurotrophic factors on SNI in rats, which may provide some clues for preclinical studies of peripheral nerve injury. However, as a result of the limited quality of included literature, this conclusion needs to be verified by a higher quality, larger sample of RCTs. Regardless, future research should focus on how to transplant cells, compare which neurotrophic factors can achieve more effective therapeutic effects, and explore the synergistic effect of various neurotrophic factors in stem cell transplantation.

## Data Availability

The English data used in this meta analysis were derived from the following resources available at [10–13,16]. The Chinese data used in this meta analysis were derived from the following resources available at: http://en.cnki.com.cn/Article_en/CJFDTOTAL-ZSZD201405008.htm, http://en.cnki.com.cn/Article_en/CJFDTOTAL-XDKF200901028.htm, http://en.cnki.com.cn/Article_en/CJFDTOTAL-ZHSY200501032.htm; [15,17–18].

## Abbreviations

BDNF: brain-derived neurotrophic factor
BMSC: bone marrow-derived mesenchymal stem cell
CI: confidence interval
CNS: central nervous system
CNTF: cholinergic neurotrophic factor
GDNF: glial-derived neurotrophic factor
hESC: human embryonic stem cell
IGF: insulin-like growth factor
MD: mean difference
NGF: nerve growth factor
NT: neurotrophic factor
PNS: peripheral nervous system
RCT: randomized controlled trial
SC: stem cell
SMD: standardized mean difference
SNFI: sciatic nerve function index
SNI: sciatic nerve injury
